# Impact of the HIV infection in Hodgkin lymphoma individuals: A protocol for systematic review and meta analysis

**DOI:** 10.1097/MD.0000000000030765

**Published:** 2022-09-30

**Authors:** Raissa Bila Cabral Fagundes, Leno Goes Delgado de Mederios, Amaxsell Thiago Barros de Souza, Maria Isabel Oliveira da Silva, Matheus Jose Barbosa Moreira, Carolina Colaço Villarrim, Irami Araújo-Filho, Kleyton Santos Medeiros

**Affiliations:** a Instituto de Ensino, Pesquisa e Inovação, Liga Contra o Câncer, Natal, Brazil; b Department of Pharmacy, Health Sciences Center, Federal University of Rio Grande do Norte (UFRN), Natal, Brazil; c Department of Medicine, Health Sciences Center, Federal University of Rio Grande do Norte (UFRN), Natal, Brazil; d Department of Haematology, Liga Contra o Câncer, Natal, Brazil; e Health Sciences Postgraduate Program, Federal University of Rio Grande do Norte (UFRN), Natal, Brazil.

**Keywords:** HIV, Hodgkin’s lymphoma, human immunodeficiency virus, lymphoma

## Abstract

Hodgkin lymphoma (HL) is a rare lymphoproliferative disorder that occurs in about 10% of all cancer cases. Human immunodeficiency virus (HIV) is associated with an increased occurrence of a wide range of cancers, including HL due to progressive immunosuppression and co-infection with oncogenic viruses. However, the aim of this systematic review is to obtain evidence about the impact of the HIV infection in HL individuals.

**Methods:**

We will obtain studies through PubMed, Embase, CINAHL, LILACS, CENTRAL, Web of Science, Scopus, Cochrane Library, and Google Scholar databases. The inclusion criteria will be observational studies (sectional, cohort, and case-control) that describe the impact of the HIV infection in HL individuals. Outcomes of interest include mortality, prevalence, causes of hospitalization, time between HIV diagnosis and HL diagnosis in days, comorbidities (systemic hypertension, diabetes mellitus, metabolic syndrome, others), T CD4 + cells/mm^3^ at HIV diagnosis and at HL diagnosis, viral load (log10 copies/mL) at HL diagnosis, and history of treatment abandon. Two reviewers, independently, will extract the data from each included study. Meta-analysis will then be carried out using fixed-effects or random-effects model, using the mean difference for continuous outcomes and the relative risk for dichotomous outcomes. Risk of bias will be assessed using the Newcastle–Ottawa Scale. The quality of evidence for each outcome will be assessed using Grading of Recommendations Assessment, Development and Evaluation methodology. Review Manager V.5.3.5 will be used for synthesis and subgroup analysis. To assess heterogeneity, we will compute the *I*^2^ statistics. Additionally, a quantitative synthesis will be performed if the included studies are sufficiently homogenous.

**Ethics and dissemination:**

This study will be a review of the published data, and thus it is not necessary to obtain ethical approval. The findings of this systematic review will be published in a peer-reviewed journal.

**PROSPERO registration number:**

CRD42021289520

## 1. Introduction

Hodgkin lymphoma (HL) is a rare lymphoproliferative disorder that occurs in about 10% of all cancer cases and whose incidence rates in developed regions such as the United States and Europe are 3 to 4 cases per 100,000.^[1–3]^ It is a largely curable condition regardless of disease stage at presentation. However, in patients with co-incidental human immunodeficiency virus (HIV) infection, it is uniquely challenging to manage,^[4]^ as it exhibits prominent epidemiological trends that differ from expected disease patterns.

HIV is associated with an increased occurrence of a wide range of cancers,^[5–8]^ including HL due to progressive immunosuppression and co-infection with oncogenic viruses. In its pathophysiological mechanism, HIV infection leads to progressive immune dysregulation and loss of immune control against the Epstein-Barr Virus, which is intrinsically related to the etiopathogenesis of HL. HIV-positive patients are 5 to 26 times more likely than HIV-negative patients to develop this lymphoproliferative neoplasm, making it the fifth most common cancer in this group.^[4,9]^

Moreover, because immunological impairment plays a decisive role in the natural history of Hodgkin disease, particularly by activating factors associated with immune escape responses and contributing to tumor progression (such as Programmed Cell Death Ligand 1 overexpression induced by Epstein-Barr Virus),^[10]^ understanding HIV comorbidity and its consequences constitute a challenge in managing these patients successfully. Of note, there’s current evidence that HIV-positive groups often present with unfavorable features at diagnosis, such as poorer performance status, extranodal disease and bone-marrow involvement.^[4,11]^ However, data on the prognosis are still conflicting and further research is necessary on the HIV-LH clinical outcomes understanding.

The introduction of highly active antiretroviral therapy (HAART) as the mainstay of HIV treatment represents a major change in the clinical course of HL and the variable impact of HAART on cancer incidence modified the proportions of tumors being diagnosed over time.^[12]^ As the immunological status of the HIV patients improves, with appropriate supportive care and widespread use of HAART, standard multidrug chemotherapy regimens (such as Adriamycin (a.k.a. Doxorubicin), Bleomycin, Vinblastine, and Dacarbazine or Bleomycin, etoposide, doxorubicin, cyclophosphamide, vincristine, procarbazine, and prednisone) can be delivered,^[13–16]^ determining 5-year PFS and overall survival rates of 69% and 78%, respectively.^[15,17]^ Based on the results of Xicoy et al,^[18–20]^ the use of the standard Adriamycin (a.k.a. Doxorubicin), Bleomycin, Vinblastine, and Dacarbazine together with HAART is feasible in advanced staged HIV-related HL and presents a tolerable long-term toxicity. Even so, despite the prognostic improvement with HAART’s introduction, cure rates remain lower than those achieved in the seronegative population.^[4]^

HIV infection and its consequences (opportunistic infections, organ dysfunction, other viral infections such as hepatitis) are associated with worse clinical outcomes, as well as drug interaction of antiretroviral regimens and chemotherapy can imply more significant toxicity or loss of efficacy in some combinations.^[4]^ In addition, supportive treatment with antivirals, antibiotics, and growth factors is usually required to reduce the risks of major adverse events and may shape the course of HL in this delicate population.^[21]^ Further, Shiels et al^[7]^ reported two-fold higher 5-year mortality for HIV-infected HL as compared to HIV-uninfected HL. Monitoring immune status seems therefore to be a decisive step in treating HIV-HL. Therefore, this protocol describes a systematic review for assessing the impact of the HIV infection in HL individuals based on updated data.

### 1.1. Research question/aim

This systematic review and meta-analysis protocol aim to clarify the clinical features and morbimortality in HIV infection patients with HL.

## 2. Methods

The systematic review will follow the guidelines of the Preferred Reporting Items for Systematic Reviews and Meta-Analyses (PRISMA) and the MOOSE (Meta-analyses of Observational Studies in Epidemiology). The protocol was designed in accordance with the Preferred Reporting Items for Systematic Reviews and Meta-Analysis guidelines extension for reporting systematic review protocols (PRISMA-P). Therefore, the review protocol was registered with the International Prospective Register of Systematic Reviews (PROSPERO), registration number (CRD42021289520).

### 2.1. Ethics

Ethical approval is not required because this review will retrieve publicly available scientific literature. Traditional dissemination strategies will be used, including open-access peer-reviewed publications, scientific presentations, and reports.

### 2.2. Inclusion criteria

This systematic review will include the following studies: observational studies (sectional, cohort, and case-control) that describe the impact of the HIV infection in HL individuals through the parameters to which they will be acquired. There will be no restrictions on the search for languages and year of publication.

### 2.3. Exclusion criteria

Will not be included in this review: case reports, meeting abstracts, review papers, and commentaries.

### 2.4. The PECOT strategy

- Population/participants: Adults (18 years old age) with HL.
- Exposure: HIV (Human Immunodeficiency Virus).
- Comparator/control: HL without HIV.
- Outcome: mortality; prevalence; causes of hospitalization (lymphoma progression, infection due to immunosuppression from chemotherapy treatment, opportunistic infection, need for dialysis therapy); time between HIV diagnosis and lymphoma diagnosis in days; T CD4 + cells/mm^3^ at HIV diagnosis and at lymphoma diagnosis; viral load at lymphoma diagnosis; history of treatment abandon.
- Types of studies: observational studies (sectional, cohort, and case-control).

### 2.5. Types of participants

Participants of the studies will be adults over 18 years old diagnosed with HL. There will be no other age or gender restriction.

### 2.6. Types of exposures

The included studies will be those describing complications in adult patients with HL HIV carries.

#### 2.6.1. Control.

The exposure group will be compared to a control of non-HIV patients with HL.

### 2.7. Types of outcome measures

The primary outcome to be evaluated will be mortality rate. The secondary outcomes to be evaluated will be prevalence, causes of hospitalization, time between HIV diagnosis and lymphoma diagnosis in days, comorbidities (systemic hypertension, diabetes mellitus, metabolic syndrome, others), T CD4 + cells/mm^3^ at HIV diagnosis and at lymphoma diagnosis and viral load (log10 copies/mL) at lymphoma diagnosis; history of treatment abandon.

### 2.8. Types of studies

Observational studies: cross-sectional, cohort, and case-control.

#### 2.8.1. Patient and public involvement.

This study is a systematic review protocol. The research will be performed with a search of literature from databases, and individual patient data will not be included. Thus, the authors will not involve patients when setting the search questions and determining the outcome measurements during the design and implementation of the study, and in the dissemination of the results.

#### 2.8.2. Search strategy.

Data from studies that evaluated the impact of the HIV infection in HL individuals will be obtained through PubMed, Embase, CINAHL, LILACS, CENTRAL, Web of Science, Scopus, Cochrane Library, and Google Scholar databases. The gray literature will be searched in databases such as OpenGrey. Articles will also be searched from the references of the retrieved studies, and the search strategy used in PubMed is presented in Table 1.

**Table 1:** Search strategy for PubMed.

PubMed search strategy	
1	Hodgkin Disease
2	Hodgkin Lymphoma
3	Adult Hodgkin Lymphoma
4	Disease, Hodgkin
5	Adult Hodgkin Lymphoma
6	Hodgkin’s Lymphoma
7	OR/ 1-6
8	HIV
9	Human Immunodeficiency Virus
10	Acquired Immune Deficiency Syndrome Virus
11	Human Immunodeficiency Viruses
12	AIDS Viruses
13	AIDS Virus
14	OR/ 8-13
15	Observational Study
16	Cohort Studies
17	Case-control Studies
18	OR/ 15-17
19	7 AND 14 AND 18

The medical subject heading (MESH) terms will be (Hodgkin Disease OR Hodgkin Lymphoma OR Adult Hodgkin Lymphoma OR Disease, Hodgkin OR Adult Hodgkin Lymphoma OR Hodgkin’s Lymphoma) AND (HIV OR Human Immunodeficiency Virus OR Acquired Immune Deficiency Syndrome Virus OR Human Immunodeficiency Viruses OR AIDS Viruses OR AIDS Virus) AND (Observational Study OR Cohort Studies OR Case-control Studies) (Table 1).

### 2.9. Data collection and analysis

#### 2.9.1. Study selection.

After exploring the databases and references, all determined articles will be exported to tool web site Rayyan in order to exclude the duplicates.^[22]^ In the first level, the titles and abstracts will be read independently by at least four reviewers (LGDM, RBCF, ATBS, and MIOS) analyzing the selected texts in order to assess compliance based on the inclusion criteria. The complete texts of these probable eligible studies will be retrieved independently and taken for eligibility by two members of the review team (LGDM and RBCF). Only studies identified by both pairs of reviewers based on the inclusion criteria will ultimately be included in the systematic review, and another reviewer (CCV) will make a final decision for inclusion in case of discrepancy.

**Figure 1.**
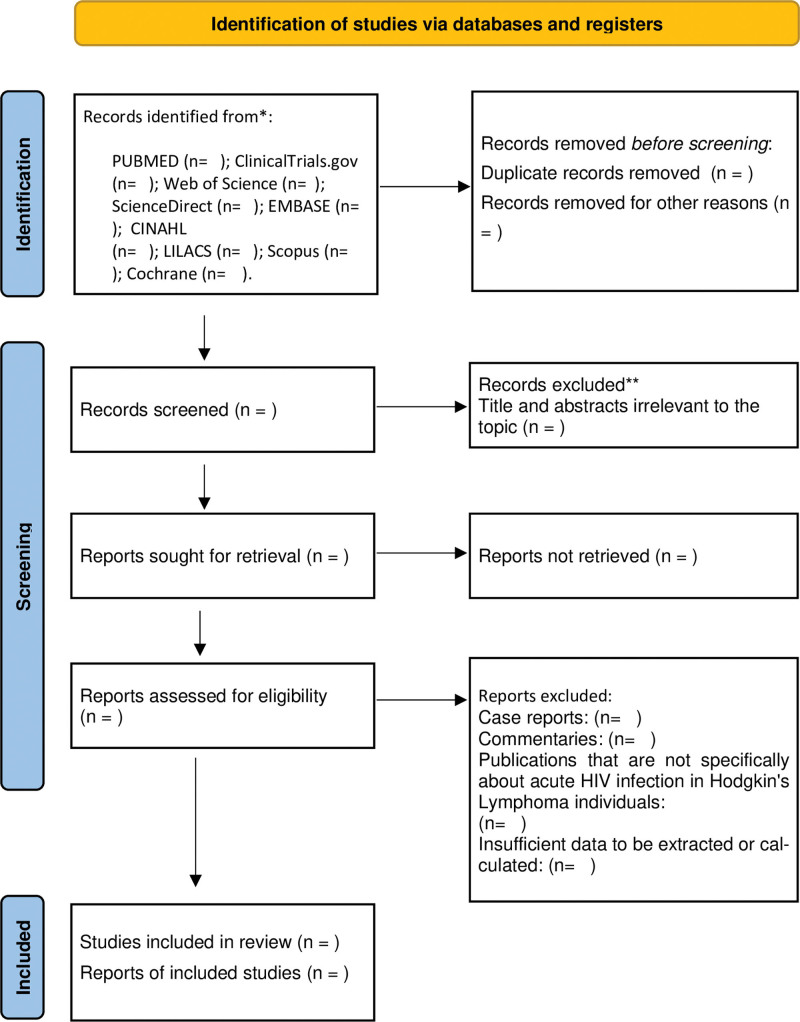
PRISMA flow diagram for systematic review and meta-analysis. HIV = human immunodeficiency virus, PRISMA = Preferred Reporting Items for Systematic Reviews and Meta-Analyses.

The results of the selection or exclusion of the studies will be summarized using the PRISMA flowchart, as shown in Figure 1.

### 2.10. Data extraction

A standardized data extraction form will be developed and tested. Two reviewers (LGDM and RBCF), independently, will extract the following data from each included study: tittle, first author, year of publication, study location, population, and sample (number of cases, number of controls, mean age, main race/ethnicity), type of study, main objectives, design and outcomes analyzed (mortality, prevalence, time of hospitalization, time between HIV diagnosis and lymphoma diagnosis in days, comorbidities, T CD4 + cells/mm^3^, viral load, and opportunistic infections). Any subsequent discrepancies will be resolved through discussion with a third review (CCV). Furthermore, participant characteristics will be extraction (e.g., mean age, gender).

#### 2.10.1. Addressing missing data.

In case of a missing data, the authors will contact the respective authors or coauthors of the article in question by telephone or e-mail. If the information will not be received, the data will be excluded from our analysis and will be covered in the discussion of the article.

#### 2.10.2. Risk of bias assessment.

Two authors (LGDM and RBCF), will independently assess the risk of bias in the eligible studies using the Newcastle–Ottawa Scale (NOS). Bias is assessed as a judgment (high, low, or unclear) for individual elements from five domains (selection, performance, attrition, reporting, and other).

#### 2.10.3. Measures of treatment effect.

We will structure the results of the systematic review with respect to the characteristics of the target population, type of primary outcomes (mortality), and secondary outcomes (prevalence, morbidity, clinical features, laboratory and imaging characteristics of adult patients with HL and HIV). The Egger funnel plot will be used to assess possible publication bias when possible.

The general prevalence of complications will be calculated using the random effects model, considering the heterogeneity between the studies included, and the Cochran *Q* test will be used to assess heterogeneity and the *I*^2^ statistic for quantification. The result of *I*^2^ ≤ 50% will be considered as low heterogeneity and a fixed effects model will be used for this case. For *I*^2^ > 50%, high heterogeneity, the random effects model will be used to calculate the prevalence ratio and 95% confidence interval (95% CI).

### 2.11. Data synthesis

A quantitative synthesis (meta-analysis) will be performed using the RevMan 5.3.5^[23]^ software using the inverse variance method with the fixed-effects or random-effects model if there is more than 50% heterogeneity between the studies. Cases where there will be insufficient data to calculate an effect estimate, a narrative synthesis will be created, describing the direction and size of the effects, along with any reported accuracy measures.

#### 2.11.1. Grading quality of evidence.

Furthermore, for grading the strength of the evidence for all outcomes from the included data, will be assessed using the Grading of Recommendations Assessment, Development and Evaluation method or an equivalent methodology will be clearly described and documented by two independent researchers (ATBS and MIOS).^[24]^ Any subsequent discrepancies will be resolved through discussion with a third reviewer (CCV).

The following items will be analyzed: exposure, comparability, sample representativeness, sample size, response rate, outcome assessment, and statistics. The Grading of Recommendations Assessment, Development and Evaluation classifies the quality of studies as low, moderate, and high. The classification of the methodological quality of the studies will be carried out considering the total number of points received: ≥ 4 for good quality and < 4 for low quality. When necessary, the divergences found will be discussed and resolved by a third author of the review (CCV).

## 3. Discussion

The incidence of HL in people living with HIV is approximately 5 times higher than HIV negative.^[7,25]^ Moreover, LH contributes to higher morbidity and mortality rates associated with HIV, as demonstrated by Olszewski et al^[26]^ in their study with 2090 patients, pointing out that 2/3 of occurrences of HIV positive with HL are connected to HIV itself, leaving only 6% complications exclusively attributable to HL. However, other studies do not confirm any significant difference between the mortality of HIV-infected and non-infected HL patients.^[5,27]^

According to Olszewski et al,^[26]^ the frequency of relapses among HL seropositive patients is associated with a T CD4 + count below 200, indicating severe immunodeficiency. Furthermore, Castillo et al^[9]^ conclude that a T CD4 + count < 200 was an independent adverse prognostic factor for disease-free survival and overall survival. However, Hentrich et al^[27]^ contradict this result, showing that low T CD4 + counts do not seem to mean unfavorable results throughout evolution.

Another important point is the late diagnosis of HL in patients with HIV, with a mean diagnosis time of 958 days. In addition, HIV patients with HL have a relatively higher T CD4 + count and a lower viral load at diagnosis than patients with non-HL.^[28]^ It has been suggested that the use of interperiod ART and the late timing of HL diagnosis are directly related. There are several particular characteristics of HL associated with HIV, including: advanced-stage disease (Ann Harbor III-IV), frequent constitutional symptoms, less favorable histology, more frequent bone marrow involvement, and worse prognosis compared to immunocompetent individuals.^[6,29]^

Interestingly, a few articles state that in HIV-positive patients, mixed cellularity is the predominant histological subtype, as opposed to HIV-negative patients, where nodular sclerosis predominates.^[9,29]^ However, other studies define nodular sclerosis as the most common subtype in both groups, with mixed cellularity being twice as common in HL seropositive patients.^[26]^ Among the studies, no higher mortality has been reported because of the change in the histological subtype found in patients with HIV and HL, except for the undefined subtype, which has a worse outcome.^[9,26,29]^

According to Olszewski et al,^[26]^ HIV-associated LH is more likely to involve extranodal tissues, particularly the bone marrow, the gastrointestinal tract, and the head and neck mucosa. Two main factors contribute to lower survival rates in seropositive patients with HL: lower treatment rates and worse prognosis for those with undetermined histological subtypes. Among the factors most associated with a lower treatment rate were: advanced age, male gender, nonwhite race, poor socioeconomic status and undetermined histological subtype.^[26]^

Regarding HIV infection and HL and results associated with morbidity and mortality, no concrete summarized evidence is available. Thus, the available scientific content must be gathered to measure the impact of HIV/LH on mortality rates. Therefore, the results of this systematic review will help to clarify the relationship between the course of HIV infection in the development of HL and the higher morbidity and mortality rate among HIV-positive patients with HL when compared to HIV-negative patients with HL. In addition, it can play an important role in improving the internal validity of future evidence in studies to be developed on the topic.

## Author contributions

**Study design:** LGDM, MJBM, RBCF, AMBS, and MIOS.

**Study supervision:** KSM and CCV.

**Manuscript writing:** LGDM, RBCF, AMBS, and MIOS.

**Critical revisions for important intellectual content:** KSM and CCV.

**Conceptualization:** Raissa Bila Cabral Fagundes, Leno Goes Delgado de Mederios, Amaxsell Thiago Barros de Souza, Maria Isabel Oliveira da Silva, Matheus Jose Barbosa Moreira, Irami Araújo-Filho.

**Methodology:** Raissa Bila Cabral Fagundes, Kleyton Santos Medeiros.

**Project administration:** Carolina Colaço Villarrim, Kleyton Santos Medeiros.

**Supervision:** Carolina Colaço Villarrim, Irami Araújo-Filho, Kleyton Santos Medeiros.

**Validation:** Raissa Bila Cabral Fagundes, Leno Goes Delgado de Mederios, Amaxsell Thiago Barros de Souza, Maria Isabel Oliveira da Silva, Matheus Jose Barbosa Moreira, Carolina Colaço Villarrim, Irami Araújo-Filho, Kleyton Santos Medeiros.

**Visualization:** Raissa Bila Cabral Fagundes, Leno Goes Delgado de Mederios, Amaxsell Thiago Barros de Souza, Maria Isabel Oliveira da Silva, Matheus Jose Barbosa Moreira, Carolina Colaço Villarrim, Irami Araújo-Filho.

**Writing – original draft:** Raissa Bila Cabral Fagundes, Leno Goes Delgado de Mederios, Amaxsell Thiago Barros de Souza, Maria Isabel Oliveira da Silva, Matheus Jose Barbosa Moreira, Carolina Colaço Villarrim, Irami Araújo-Filho, Kleyton Santos Medeiros.

**Writing – review & editing:** Matheus Jose Barbosa Moreira, Carolina Colaço Villarrim, Irami Araújo-Filho, Kleyton Santos Medeiros.
